# Na_2_MoO_2_As_2_O_7_

**DOI:** 10.1107/S1600536812048167

**Published:** 2012-11-30

**Authors:** Raja Jouini, Mohamed Faouzi Zid, Ahmed Driss

**Affiliations:** a Laboratoire de Matériaux et Cristallochimie, Faculté des Sciences de Tunis, Université de Tunis ElManar, 2092 ElManar II Tunis, Tunisia

## Abstract

Disodium molybdenum dioxide diarsenate, Na_2_MoO_2_As_2_O_7_, has been synthesized by a solid-state reaction. The structure is built up from MoAs_2_O_12_ linear units sharing corners to form a three-dimensional framework containing tunnels running along the *a-*axis direction in which the Na^+^ cations are located. In this framework, the As^V^ atoms are tetra­hedrally coordinated and form an As_2_O_7_ group. The Mo^VI^ atom is displaced from the center of an octa­hedron of O atoms. Two Na^+^ cations are disordered about inversion centres. Structural relationships between different compounds: *A*_2_MoO_2_As_2_O_7_ (*A* = K, Rb), *AM*OP_2_O_7_ (*A* = Na, K, Rb; *M* = Mo, Nb) and MoP_2_O_7_ are discussed.

## Related literature

For background to the physico-chemical properties of related compounds, see: Daidouh *et al.* (1997 ▶); Troup & Clearfield (1977 ▶); Guesdon *et al.* (1990 ▶). For details of structurally related compounds, see: Averbuch-Pouchot (1988 ▶, 1989 ▶); Zid *et al.* (1998 ▶, 2003 ▶); Ledain *et al.* (1996 ▶); Gueho *et al.* (1992 ▶); Fakhfakh *et al.* (1994 ▶); Linde *et al.* (1980 ▶); Leclaire *et al.* (1988 ▶). For the preparation, see: Zid & Jouini (1996 ▶); Zid *et al.* (1997 ▶, 1998 ▶); Hajji *et al.* (2004 ▶); Hajji & Zid (2006 ▶); Jouini *et al.* (2012 ▶); Ben Hlila *et al.* (2009 ▶). For bond-valence sums, see: Brown & Altermatt (1985 ▶).

## Experimental

### 

#### Crystal data

Na_2_MoO_2_As_2_O_7_
*M* *_r_* = 435.76
Monoclinic, 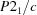

*a* = 7.1130 (7) Å
*b* = 12.056 (2) Å
*c* = 9.8030 (9) Å
β = 100.48 (2)°
*V* = 826.63 (18) Å^3^
*Z* = 4
Mo *K*α radiation
μ = 9.66 mm^−1^
*T* = 298 K
0.56 × 0.32 × 0.21 mm

#### Data collection

Enraf–Nonius CAD-4 diffractometer
Absorption correction: ψ scan (North *et al.*, 1968 ▶) *T*_min_ = 0.037, *T*_max_ = 0.134
2280 measured reflections
1790 independent reflections
1711 reflections with *I* > 2σ(*I*)
*R*_int_ = 0.019
2 standard reflections every 120 min intensity decay: 1.2%

#### Refinement

*R*[*F* ^2^ > 2σ(*F* ^2^)] = 0.022
*wR*(*F* ^2^) = 0.058
*S* = 1.25
1790 reflections
137 parameters
Δρ_max_ = 0.83 e Å^−3^
Δρ_min_ = −0.75 e Å^−3^

### 

Data collection: *CAD-4 EXPRESS* (Duisenberg, 1992 ▶; Macíček & Yordanov, 1992 ▶); cell refinement: *CAD-4 EXPRESS*; data reduction: *XCAD4* (Harms & Wocadlo, 1995 ▶); program(s) used to solve structure: *SHELXS97* (Sheldrick, 2008 ▶); program(s) used to refine structure: *SHELXL97* (Sheldrick, 2008 ▶); molecular graphics: *DIAMOND* (Brandenburg, 1998 ▶); software used to prepare material for publication: *WinGX* (Farrugia, 2012 ▶).

## Supplementary Material

Crystal structure: contains datablock(s) I, global. DOI: 10.1107/S1600536812048167/br2214sup1.cif

Structure factors: contains datablock(s) I. DOI: 10.1107/S1600536812048167/br2214Isup2.hkl

Additional supplementary materials:  crystallographic information; 3D view; checkCIF report

## Figures and Tables

**Fig. 1.**
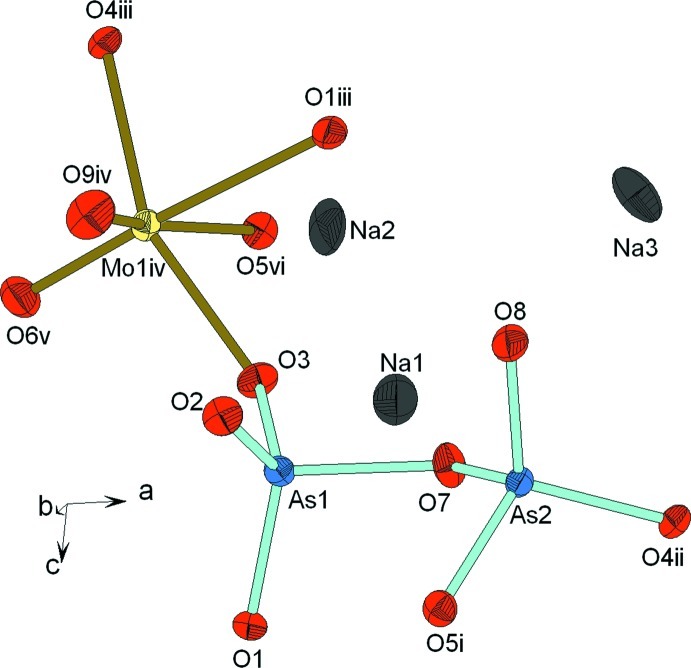
: Unité asymétrique dans Na2MoO2As2O7. Les ellipsoïdes ont été définis avec 50% de probabilités. [codes de symétrie:(i) x, 1 + y, z; (ii) 1 + x, y, z; (iii) x, 3/2 - y, -1/2 + z; (iv) x, 3/2 - y, 1/2 + z; (v) -1 + x, 3/2 - y, 1/2 + z; (vi) 1 - x, 1/2 + y, 3/2 - z].

**Fig. 2.**
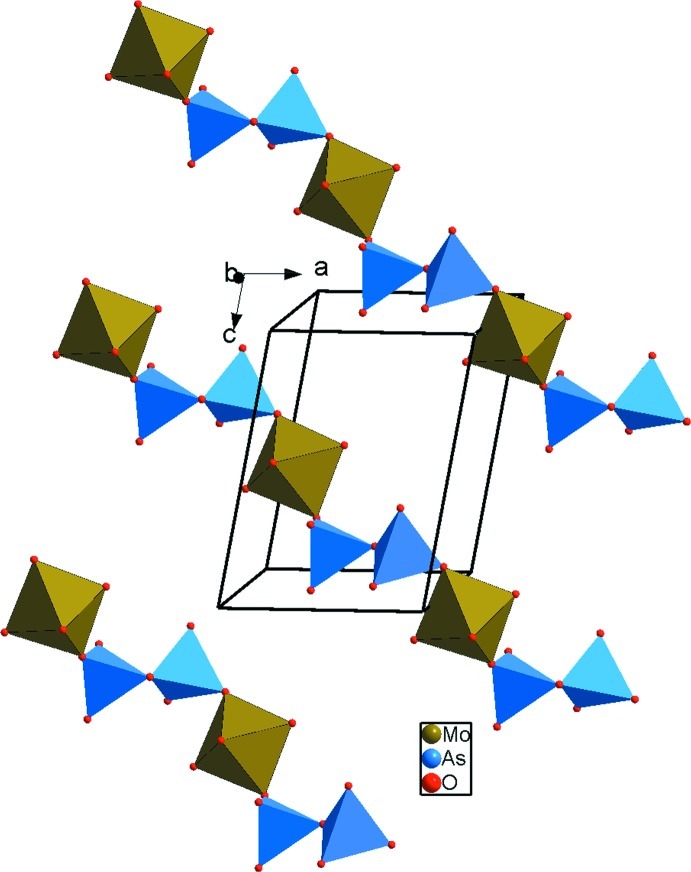
: Projection de chaînes de type MoAs2O11 selon la direction [10–1].

**Fig. 3.**
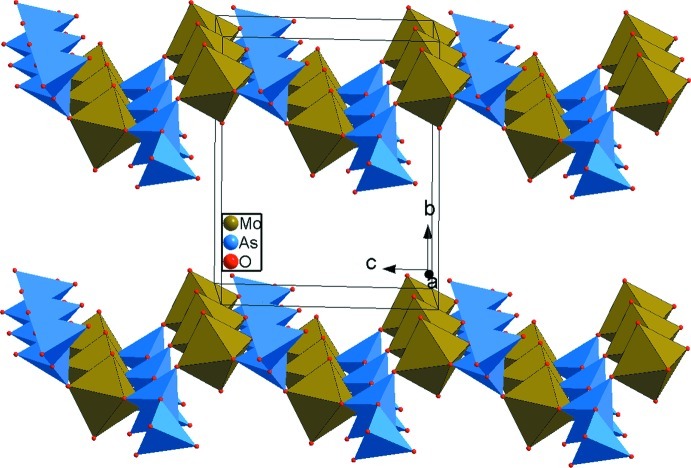
: Disposition de couches ondulées de type MoAs2O10 selon c.

**Fig. 4.**
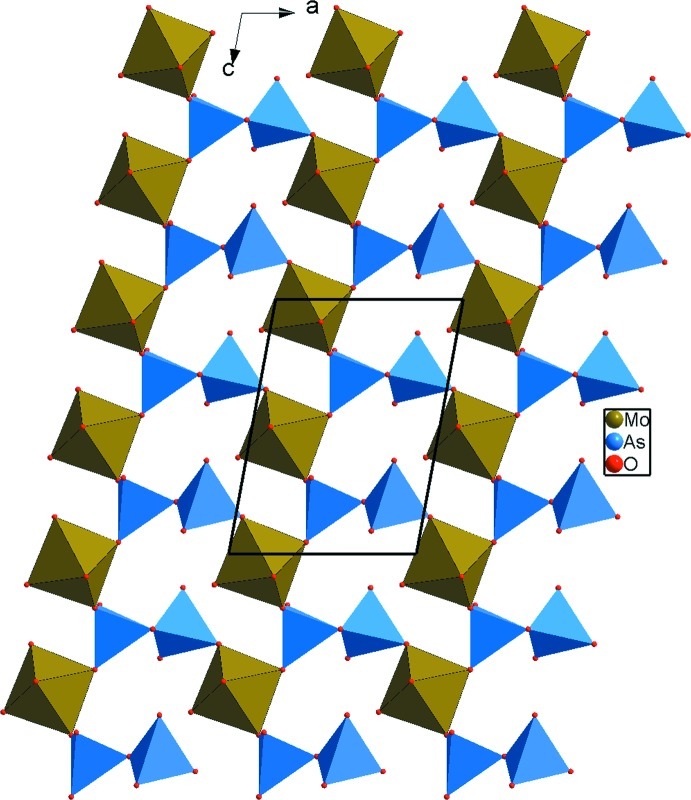
: Projection d'une couche de type MoAs2O10 dans le plan ac.

**Fig. 5.**
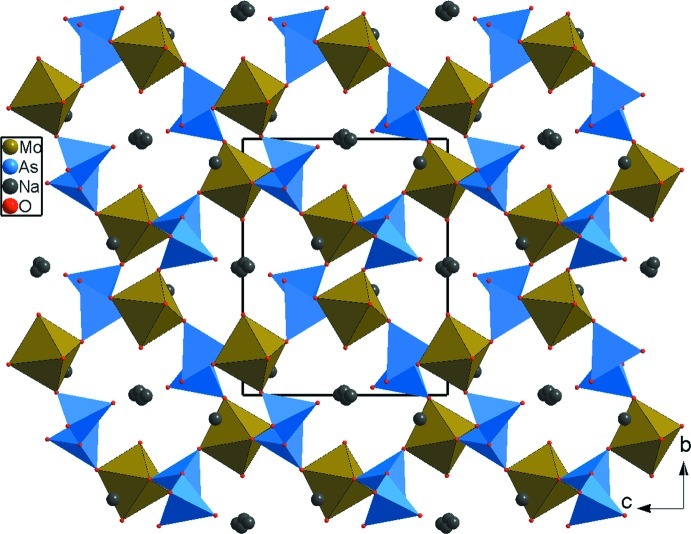
: Projection de la structure de Na2MoO2As2O7 selon a, mettant en évidence les canaux où logent les cations Na+.

## supplementary crystallographic information

### Comment

*L*'élaboration de nouveaux matériaux à charpentes mixtes constituées d'octaèdres MO_6_ (*M*= métal de transition) et de tétraèdres XO_4_ (*X*= P, As) est un domaine d'intense activité. En effet, ces matériaux peuvent être potentiellement des conducteurs ioniques (Daidouh *et al.*, 1997), des échangeurs d'ions (Troup & Clearfield, 1977) ou bien comme produits d'intercalation (Guesdon *et al.*, 1990).

C'est dans ce cadre que nous avons exploré les systèmes A—Mo—As—O (A= alcalin, argent) dans lesquels nous avons précédemment caractérisé les phases suivantes: K_2_MoO_2_As_2_O_7_ (Zid & Jouini, 1996), AgMoO_2_AsO_4_ (Hajji & Zid, 2006), Rb_2_MoO_2_As_2_O_7_ (Zid *et al.*, 1998) et Na_2_(MoO_2_)_3_(As_2_O_7_)_2_ (Jouini *et al.*, 2012).

Une nouvelle phase de formulation Na_2_MoO_2_As_2_O_7_ a été synthétisée par réaction à l'état solide. *L*'unité *asym*étrique dans la structure renferme un octaèdre MoO_6_ et un groupement diarséniate As_2_O_7_ reliés par mise en commun d'un sommet formant ainsi l'unité MoAs_2_O_12_ (Fig. 1). Ces dernières s'associent par mise en commun de sommets pour établir des chaînes infinies MoAs_2_O_11_ disposées selon la direction [10–1] (Fig. 2). *L*'association de ces chaînes est assurée par partage de sommets entre les polyèdres de nature différente conduisant à des couches ondulées MoAs_2_O_10_ (Fig. 3). Ces dernières présentent dans le plan *ac* des rangées d'octaèdres MoO_6_ entre lesquelles sont disposés les groupements diarséniates (Fig. 4). La cohésion de ces feuillets, assurée par formation de ponts mixtes Mo—O—As conduit à une structure tridimensionnelle possédant de larges canaux où logent les cations Na^+^ (Fig. 5).

L'examen des facteurs géométriques dans la structure montre qu'ils sont conformes à ceux rencontrés dans la littérature (Zid *et al.*, 1997; Hajji *et al.*, 2004; Hajji & Zid, 2006; Ben Hlila *et al.*, 2009). De plus, l'utilisation de la méthode BVS, pour le calcul des différentes valences des liaisons, utilisant la formule empirique de Brown (Brown & Altermatt, 1985) vérifie bien les valeurs attendues de charges des ions: Mo1(5,955), As1(4,790), As2(4,913), Na1(0,900), Na2(0,815) et Na3(0,899).

La comparaison de la structure de Na_2_MoO_2_As_2_O_7_ avec celles rencontrées dans la littérature et de formulation analogue A_2_MoO_2_P_2_O_7_ (A=Cs (Averbuch-Pouchot, 1988); A=NH_4_ (Averbuch-Pouchot, 1989); A=K (Zid *et al.*, 2003)) et A_2_MoO_2_As_2_O_7_ (A=K (Zid & Jouini, 1996); A=Rb (Zid *et al.*, 1998)) montre que les diphosphates présentent des charpentes unidimensionnelles (one-dimensional) qui sont construites moyennant des unités cycliques de formulation MoP_2_O_11_ dans lesquelles un octaèdre MoO_6_ partage deux de ses sommets avec le même groupement P_2_O_7_. En revanche, une relation structurale peut être mise en évidence d'une part entre le diarséniate étudié et ceux de type A_2_MoO_2_As_2_O_7_ (A=K (Zid & Jouini, 1996); A=Rb (Zid *et al.*, 1998)) et d'autre part avec les composés de formulation différente AMOP_2_O_7_ (A=Na, *M*=Mo (Ledain *et al.*, 1996); A=K, *M*=Mo (Gueho *et al.*, 1992); A=Rb, *M*=Nb (Fakhfakh *et al.*, 1994) et A=K, *M*=Nb (Linde *et al.*, 1980)). En effet, leurs charpentes renferment le même type d'unité linéaire *MX*_2_O_12_ (*M*=Mo, Nb et *X*= P, As). Cependant ces structures diffèrent par l'organisation de ces unités. Elles s'associent par mise en commun de sommets pour former des chaînes parallèles qui se lient au moyen de ponts mixtes Mo—O—As pour conduire à des charpentes (two-dimensional) dans les diarséniates K_2_MoO_2_As_2_O_7_ et Rb_2_MoO_2_As_2_O_7_. Alors qu'elles s'organisent en chaînes ondulées dans les diphosphates AMoOP_2_O_7_ (A=Na, K) et ANbOP_2_O_7_ (A=K, Rb). Ces chaînes s'associent par formation de ponts mixtes M—O—P (*M*=Mo, Nb) pour conduire à des charpentes tridimensionnelles (three-dimensional). Ces unités linéaires Mo*X*_2_O_12_ (*X*= P, As) forment par mise en commun de tous les sommets entre polyèdres de nature différente des charpentes (three-dimensional) donnant lieu à un oxyde mixte de formulation MoP_2_O_7_ (Leclaire *et al.*, 1988). La compensation de la charge dans ce dernier a été assurée d'une part par élimination du cation et d'autre part réduction de l'ion molybdenium.

### Experimental

Les cristaux relatifs à la phase Na_2_MoO_2_As_2_O_7_ ont été obtenus à partir des réactifs solides Na_2_CO_3_ (Prolabo, 27778), (NH_4_)_2_Mo_4_O_13_ (Fluka, 69858) et NH_4_H_2_AsO_4_ (préparé au laboratoire, ASTM 01–775) pris dans les proportions: Na:Mo:As=2:1:2. Le mélange a été finement broyé, puis préchauffé à l'air jusqu'à 623 K pendant 24 heures en vue d'éliminer les composés volatils. Après refroidissement et broyage, la préparation est portée, proche de la fusion pour favoriser la germination et la croissance des cristaux, à 838 K pendant une semaine. Le résidu final a subi en premier un refroidissement lent (5°/demi journée, à 778 K) puis un second rapide (50°/h) jusqu'à la température ambiante. Par lavage à l'eau chaude des cristaux de couleur jaunâtre de qualité et de taille suffisante ont été séparés pour analyse par DRX.

### Refinement

Les deux ions Na2 et Na3 sont situés sur des positions *sp*éciales. Ayant des agitations thermiques assez élevées, leurs coordonnées atomiques ont été affinées et conduit statistiquement vers des positions très proches respectivement de leurs positions initiales. En effet, l'affinement final des facteurs thermiques anisotropes conduit à des ellipsoïdes bien définis. De plus, les densités d'électrons maximums et minimums restant dans la Fourier différence, sont acceptables et sont situées respectivement à 0,96 Å de O5 et à 0,96 Å de As2.

### Crystal data

**Table:**

Na_2_MoO_2_As_2_O_7_	*F*(000) = 808
*M**_r_* = 435.76	*D*_x_ = 3.501 Mg m^−^^3^
Monoclinic, *P*2_1_/*c*	Mo *K*α radiation, λ = 0.71073 Å
Hall symbol: -P 2ybc	Cell parameters from 25 reflections
*a* = 7.1130 (7) Å	θ = 10–15°
*b* = 12.056 (2) Å	µ = 9.66 mm^−^^1^
*c* = 9.8030 (9) Å	*T* = 298 K
β = 100.48 (2)°	Prism, yellow
*V* = 826.63 (18) Å^3^	0.56 × 0.32 × 0.21 mm
*Z* = 4	

### Data collection

**Table:**

Enraf–Nonius CAD-4 diffractometer	1711 reflections with *I* > 2σ(*I*)
Radiation source: fine-focus sealed tube	*R*_int_ = 0.019
Graphite monochromator	θ_max_ = 27.0°, θ_min_ = 2.7°
ω/2θ scans	*h* = −9→9
Absorption correction: ψ scan (North *et al.*, 1968)	*k* = −1→15
*T*_min_ = 0.037, *T*_max_ = 0.134	*l* = −12→1
2280 measured reflections	2 standard reflections every 120 min
1790 independent reflections	intensity decay: 1.2%

### Refinement

**Table:**

Refinement on *F*^2^	Primary atom site location: structure-invariant direct methods
Least-squares matrix: full	Secondary atom site location: difference Fourier map
*R*[*F*^2^ > 2σ(*F*^2^)] = 0.022	*w* = 1/[σ^2^(*F*_o_^2^) + (0.0229*P*)^2^ + 2.4476*P*] where *P* = (*F*_o_^2^ + 2*F*_c_^2^)/3
*wR*(*F*^2^) = 0.058	(Δ/σ)_max_ = 0.001
*S* = 1.25	Δρ_max_ = 0.83 e Å^−^^3^
1790 reflections	Δρ_min_ = −0.75 e Å^−^^3^
137 parameters	Extinction correction: *SHELXL97* (Sheldrick, 2008), Fc^*^=kFc[1+0.001xFc^2^λ^3^/sin(2θ)]^-1/4^
0 restraints	Extinction coefficient: 0.0083 (4)

### Special details

**Table:**

Geometry. All e.s.d.'s (except the e.s.d. in the dihedral angle between two l.s. planes) are estimated using the full covariance matrix. The cell e.s.d.'s are taken into account individually in the estimation of e.s.d.'s in distances, angles and torsion angles; correlations between e.s.d.'s in cell parameters are only used when they are defined by crystal symmetry. An approximate (isotropic) treatment of cell e.s.d.'s is used for estimating e.s.d.'s involving l.s. planes.
Refinement. Refinement of *F*^2^ against ALL reflections. The weighted *R*-factor *wR* and goodness of fit *S* are based on *F*^2^, conventional *R*-factors *R* are based on *F*, with *F* set to zero for negative *F*^2^. The threshold expression of *F*^2^ > σ(*F*^2^) is used only for calculating *R*-factors(gt) *etc*. and is not relevant to the choice of reflections for refinement. *R*-factors based on *F*^2^ are statistically about twice as large as those based on *F*, and *R*- factors based on ALL data will be even larger.

### Fractional atomic coordinates and isotropic or equivalent isotropic displacement parameters (Å^2^)

**Table:**

	*x*	*y*	*z*	*U*_iso_*/*U*_eq_	Occ. (<1)
Mo1	0.14180 (4)	0.82040 (3)	0.04354 (3)	0.01031 (11)	
As1	0.42263 (5)	0.83066 (3)	0.78808 (4)	0.01048 (12)	
As2	0.81207 (5)	0.92725 (3)	0.79392 (4)	0.00972 (12)	
Na1	0.3687 (3)	0.09290 (17)	0.8660 (2)	0.0314 (4)	
Na2	0.504 (10)	0.986 (5)	0.494 (7)	0.047 (7)	0.50
Na3	0.9657 (8)	0.9978 (6)	0.4736 (5)	0.0263 (13)	0.50
O1	0.4007 (4)	0.8279 (2)	0.9549 (3)	0.0166 (6)	
O2	0.3637 (4)	0.9467 (2)	0.7019 (3)	0.0165 (6)	
O3	0.3405 (4)	0.7092 (2)	0.7129 (3)	0.0151 (5)	
O4	0.0359 (4)	0.8798 (2)	0.8550 (3)	0.0142 (5)	
O5	0.7368 (4)	0.0110 (2)	0.9099 (3)	0.0136 (5)	
O6	0.9545 (4)	0.8561 (3)	0.1218 (3)	0.0212 (6)	
O7	0.6693 (4)	0.8070 (2)	0.7961 (3)	0.0158 (6)	
O8	0.7899 (4)	0.9695 (3)	0.6338 (3)	0.0198 (6)	
O9	0.0977 (5)	0.6856 (2)	0.0018 (3)	0.0237 (7)	

### Atomic displacement parameters (Å^2^)

**Table:**

	*U*^11^	*U*^22^	*U*^33^	*U*^12^	*U*^13^	*U*^23^
Mo1	0.00956 (17)	0.00924 (17)	0.01180 (17)	0.00032 (11)	0.00105 (12)	0.00149 (11)
As1	0.0103 (2)	0.01014 (19)	0.01110 (19)	−0.00184 (13)	0.00220 (14)	−0.00231 (13)
As2	0.00852 (18)	0.01071 (19)	0.00969 (18)	−0.00010 (13)	0.00098 (13)	0.00109 (13)
Na1	0.0221 (9)	0.0364 (10)	0.0343 (10)	0.0016 (8)	0.0013 (8)	−0.0166 (9)
Na2	0.023 (4)	0.09 (2)	0.026 (9)	0.028 (13)	0.005 (5)	0.027 (13)
Na3	0.032 (4)	0.0183 (15)	0.035 (4)	0.002 (3)	0.022 (3)	0.004 (3)
O1	0.0135 (13)	0.0258 (15)	0.0105 (12)	0.0011 (11)	0.0024 (10)	−0.0042 (11)
O2	0.0182 (14)	0.0141 (13)	0.0169 (13)	0.0001 (11)	0.0023 (11)	0.0011 (11)
O3	0.0167 (13)	0.0130 (13)	0.0144 (12)	−0.0048 (11)	−0.0001 (10)	−0.0022 (11)
O4	0.0113 (12)	0.0185 (14)	0.0116 (12)	0.0022 (10)	−0.0010 (10)	0.0018 (10)
O5	0.0139 (12)	0.0115 (12)	0.0153 (12)	0.0018 (10)	0.0023 (10)	−0.0009 (10)
O6	0.0173 (14)	0.0267 (15)	0.0211 (14)	0.0035 (12)	0.0073 (11)	0.0058 (12)
O7	0.0122 (13)	0.0116 (12)	0.0243 (14)	−0.0022 (10)	0.0056 (11)	−0.0031 (11)
O8	0.0152 (13)	0.0312 (16)	0.0129 (13)	0.0042 (12)	0.0024 (10)	0.0085 (12)
O9	0.0277 (16)	0.0133 (14)	0.0277 (16)	−0.0043 (12)	−0.0014 (13)	0.0008 (12)

### Geometric parameters (Å, º)

**Table:**

Mo1—O9	1.692 (3)	Na1—O2^viii^	2.382 (3)
Mo1—O6^i^	1.709 (3)	Na1—O6^iv^	2.403 (3)
Mo1—O4^ii^	1.997 (3)	Na1—O3^ix^	2.725 (3)
Mo1—O3^iii^	2.006 (3)	Na1—O5^x^	2.749 (3)
Mo1—O1^ii^	2.177 (3)	Na1—O5	2.759 (3)
Mo1—O5^iv^	2.223 (3)	Na1—O7^ix^	3.017 (3)
As1—O2	1.649 (3)	Na2—O8	2.25 (7)
As1—O1	1.671 (3)	Na2—O8^xi^	2.29 (7)
As1—O3	1.695 (3)	Na2—O2^xi^	2.43 (7)
As1—O7	1.765 (3)	Na2—O2	2.47 (7)
As2—O8	1.630 (3)	Na3—O8	2.204 (8)
As2—O5^v^	1.679 (3)	Na3—O8^xii^	2.226 (7)
As2—O4^vi^	1.695 (3)	Na3—O9^xiii^	2.329 (8)
As2—O7	1.772 (3)	Na3—O9^xiv^	2.398 (8)
Na1—O1^vii^	2.374 (3)	Na3—O2^xi^	2.726 (5)
			
O9—Mo1—O6^i^	103.00 (16)	O6^iv^—Na1—O3^ix^	131.68 (13)
O9—Mo1—O4^ii^	95.67 (13)	O1^vii^—Na1—O5^x^	81.05 (10)
O6^i^—Mo1—O4^ii^	97.55 (13)	O2^viii^—Na1—O5^x^	103.30 (12)
O9—Mo1—O3^iii^	95.80 (14)	O6^iv^—Na1—O5^x^	70.98 (10)
O6^i^—Mo1—O3^iii^	99.37 (13)	O3^ix^—Na1—O5^x^	142.44 (11)
O4^ii^—Mo1—O3^iii^	156.85 (11)	O1^vii^—Na1—O5	61.30 (10)
O9—Mo1—O1^ii^	94.41 (14)	O2^viii^—Na1—O5	74.79 (10)
O6^i^—Mo1—O1^ii^	162.57 (13)	O6^iv^—Na1—O5	166.71 (13)
O4^ii^—Mo1—O1^ii^	79.62 (11)	O3^ix^—Na1—O5	58.68 (9)
O3^iii^—Mo1—O1^ii^	79.51 (11)	O5^x^—Na1—O5	95.82 (10)
O9—Mo1—O5^iv^	167.51 (13)	O1^vii^—Na1—O7^ix^	91.68 (11)
O6^i^—Mo1—O5^iv^	89.03 (13)	O2^viii^—Na1—O7^ix^	106.75 (11)
O4^ii^—Mo1—O5^iv^	85.92 (10)	O6^iv^—Na1—O7^ix^	79.20 (11)
O3^iii^—Mo1—O5^iv^	78.69 (11)	O3^ix^—Na1—O7^ix^	53.88 (8)
O1^ii^—Mo1—O5^iv^	73.65 (10)	O5^x^—Na1—O7^ix^	143.06 (10)
O2—As1—O1	117.26 (14)	O5—Na1—O7^ix^	112.34 (10)
O2—As1—O3	118.35 (14)	O8—Na2—O8^xi^	171 (3)
O1—As1—O3	108.81 (14)	O8—Na2—O2^xi^	94 (3)
O2—As1—O7	108.54 (13)	O8^xi^—Na2—O2^xi^	86 (2)
O1—As1—O7	102.82 (14)	O8—Na2—O2	86 (2)
O3—As1—O7	98.11 (13)	O8^xi^—Na2—O2	92 (2)
O8—As2—O5^v^	118.50 (14)	O2^xi^—Na2—O2	171 (2)
O8—As2—O4^vi^	111.47 (13)	O8—Na3—O8^xii^	163.26 (19)
O5^v^—As2—O4^vi^	110.97 (13)	O8—Na3—O9^xiii^	86.1 (3)
O8—As2—O7	108.19 (15)	O8^xii^—Na3—O9^xiii^	93.4 (3)
O5^v^—As2—O7	103.27 (13)	O8—Na3—O9^xiv^	92.1 (3)
O4^vi^—As2—O7	102.88 (13)	O8^xii^—Na3—O9^xiv^	83.9 (3)
O1^vii^—Na1—O2^viii^	136.07 (13)	O9^xiii^—Na3—O9^xiv^	164.40 (18)
O1^vii^—Na1—O6^iv^	113.49 (12)	O8—Na3—O2^xi^	87.3 (2)
O2^viii^—Na1—O6^iv^	109.05 (13)	O8^xii^—Na3—O2^xi^	108.3 (2)
O1^vii^—Na1—O3^ix^	62.93 (10)	O9^xiii^—Na3—O2^xi^	70.52 (19)
O2^viii^—Na1—O3^ix^	96.18 (11)	O9^xiv^—Na3—O2^xi^	124.9 (2)

Symmetry codes: (i) *x*−1, *y*, *z*; (ii) *x*, *y*, *z*−1; (iii) *x*, −*y*+3/2, *z*−1/2; (iv) −*x*+1, −*y*+1, −*z*+1; (v) *x*, *y*+1, *z*; (vi) *x*+1, *y*, *z*; (vii) −*x*+1, −*y*+1, −*z*+2; (viii) *x*, *y*−1, *z*; (ix) −*x*+1, *y*−1/2, −*z*+3/2; (x) −*x*+1, −*y*, −*z*+2; (xi) −*x*+1, −*y*+2, −*z*+1; (xii) −*x*+2, −*y*+2, −*z*+1; (xiii) −*x*+1, *y*+1/2, −*z*+1/2; (xiv) *x*+1, −*y*+3/2, *z*+1/2.
